# COVID-19 vaccination and HIV transmission among persons who inject drugs during the first two years of the COVID-19 pandemic in New York City

**DOI:** 10.1186/s12954-023-00791-0

**Published:** 2023-05-03

**Authors:** Don C. Des Jarlais, Chenziheng Allen Weng, Jonathan Feelemyer, Courtney McKnight

**Affiliations:** School of Global Public Health, New York University, 708 Broadway, 7th Floor, NY 10003 New York, USA

**Keywords:** Persons who inject drugs (PWID), New York City, HIV, Incidence, COVID-19, Vaccination

## Abstract

**Background:**

To examine COVID-19 vaccination and HIV transmission among persons who inject drugs (PWID) during the COVID-19 pandemic (2020–2022) in New York City (NYC).

**Methods:**

Two hundred and seventy five PWID were recruited from October 2021 to September 2022. A structured questionnaire was used to measure demographics, drug use behaviors, overdose experiences, substance use treatment history, COVID-19 infection, vaccination, and attitudes. Serum samples were collected for HIV, HCV, and SARS-CoV-2 (COVID-19) antibody testing.

**Results:**

Participants were: 71% male, the mean age was 49 (SD 11), 81% reported at least one COVID-19 immunization, 76% were fully vaccinated and 64% of the unvaccinated had antibodies for COVID-19. Self-reported injection risk behaviors were very low. HIV seroprevalence was 7%. Eighty-nine percent of the HIV seropositive respondents reported knowing they were HIV seropositive and being on antiretroviral therapy prior to the COVID-19 pandemic. There were two likely seroconversions in 518.83 person-years at risk from the March 2020 start of the pandemic to the times of interviews, for an estimated incidence rate of 0.39/100 person-years, 95% Poisson CI 0.05–1.39/100 person-years.

**Conclusions:**

There is concern that the COVID-19 pandemic disruptions to HIV prevention services and the psychological stress of the pandemic may lead to increased risk behavior and increased HIV transmission. These data indicate adaptive/resilient behaviors in both obtaining COVID-19 vaccination and maintaining a low rate of HIV transmission among this sample of PWID during the first two years of the COVID-19 pandemic in NYC.

## Introduction

The COVID-19 pandemic and its associated behavior restrictions (“lockdowns”) have posed multiple severe threats to the health of persons who use drugs (PWUD). First is the danger of SARS-CoV-2/COVID-19 infection itself. Many PWUD have “underlying conditions” that are associated with developing more severe COVID-19 disease, e.g., cardiovascular disease, pulmonary disease, diabetes, and HIV infection [1]. COVID-19 vaccines would provide the greatest means of protecting against severe COVID-19 infection among PWUD.

The COVID-19 pandemic also disrupted many HIV prevention and treatment services [2–5] leading to a concern that the pandemic may facilitate outbreaks of HIV infection, particularly among persons who inject drugs (PWID). There are reports of increased HIV risk behaviors and of several outbreaks of HIV infection among PWID during the pandemic [6–8]. The best method of trying to prevent such outbreaks would be to rapidly restore full HIV prevention and care services, including syringe service programs (SSP), treatment programs for substance use disorders, and antiretroviral treatment (ART) for HIV infection. Large-scale vaccination of PWUD for COVID-19, including booster immunizations, may reduce the likelihood of future disruption of in-person HIV prevention and care services.

New York City (NYC) was an epicenter of COVID-19 during the early part of the pandemic [9] and also imposed strict “pause/lockdown” measures [10]. Once COVID-19 vaccines were authorized for emergency use, the NYC government made a strong effort to promote vaccination, including an extensive public service information campaign [11] and focused efforts to reach persons considered to be a high risk for COVID-19 disease [12]. The city and state health departments, treatment providers, and local community-based organizations also made intensive efforts to adapt to the COVID-19 pandemic restrictions and restore the HIV prevention programs that had been initially disrupted. Syringe Services Programs (SSP) were declared to be “essential services” that were to continue during the pandemic. Immediately after the “pause/lockdown,” SSPs reported a decline in the numbers of visits (encounters) and in the numbers of individuals attending the programs. However, the SSPs responded by increasing the numbers of sterile syringes distributed per encounter such that the number of syringes distributed actually increased during the first 6 months of the pause/lockdown, and then returned to pre-pandemic levels [13]. Because of social distancing requirements, HIV and HCV antibody testing were the services most likely to be interrupted, and the most difficult to restore [14].

Methadone programs reduced the requirements for in-person counseling and increased “take home” medication—to 28 days of medication for “stable” patients and 14 days for “less stable” patients [15]. A home delivery service of methadone for persons with COVID-19 or other reasons why they would not be able to attend a clinic was established [15]. The City Health and Hospitals Corporation opened a remote/telehealth clinic for providing buprenorphine treatment [16].

During the first year of the pandemic, there was a stable level of viral suppression among PWID; among those that had been in HIV medical care (defined by having at least one viral load test), viral suppression was 83% in 2019 and 81% in 2020; among those in sustained HIV medical care (defined as having at least two viral load tests in the previous 14 months) viral suppression was 61% in 2019 and 62% in 2020 [17, 18].

We do not want to minimize the difficulties created by social distancing, stay at home guidance, isolation and quarantines, and the shortages of personal protective equipment at the start of the COVID-19 pandemic/lockdown in New York City, but the SSP’s and other prevention and treatment programs and their participants rapidly adapted to the new circumstances.

We are aware of one early report on HIV risk behavior among PWID in NYC during the COVID-19 pandemic [2]. Aponte-Melendez et al. conducted a cohort study in which they compared HIV risk behavior among PWID interviewed in the year before the COVID-19 pause/lockdown to PWID interviewed during the first year of the pause/lockdown. They found evidence for increased re-use of own syringes, increased sharing of filters, cookers, rinse water and water containers. However, none of their subjects reported receptive sharing of syringes during the first year of the pandemic.

Acquiring COVID-19 infection and or HIV infection were clearly two major threats to PWID health during the pandemic. We report here on COVID-19 vaccinations, safer injecting, and HIV transmission during the first two years of the COVID-19 pandemic (March 2020 to May 2022) among PWID in NYC. This period covers the initial disruption of services and the adaptation/restoration of services as well as the first year of the availability of COVID-19 vaccines.

## Methods

### Subject recruitment and data collection

Beginning in October 2021, study participants were recruited using an adapted version of respondent-driven sampling (RDS). An initial set of 14 seeds were recruited from public parks and areas adjacent to SSPs and methadone maintenance treatment programs in Manhattan (locations where PWID were known to congregate). Seeds were selected to reflect the demographic characteristics of PWID in NYC with respect to age, sex, and race/ethnicity. However, a combination of lagging peer referral via RDS coupons and suspension of study activities due to the Omicron surge of COVID-19 in NYC led to disruptions in peer referral. To increase recruitment, we employed a variety of methods, including staff recruitment of additional seeds, an increase in the number of referral coupons from 3 to 6 for existing participants and allowances for people who lost referral coupons.

Study eligibility criteria included: at least 18 years of age, reported injection of heroin, fentanyl, cocaine, crack, or methamphetamine within the previous 30 days, able to speak and understand English, ability to give informed consent, and planning on residing in the NYC-metro area for the next 6 months. Eligible PWID were enrolled in a 6-month serial cohort study consisting of two in-person appointments at baseline and six months. Each visit included a survey, urine toxicology screening, and blood draw for HIV, HCV and SARS-CoV-2 antibody testing. At each visit, experienced interviewers conducted one-on-one, computer-assisted structured interviews which lasted approximately 30 min. Questionnaire Development System (QDS) software (Nova Research Company, Bethesda, MD, USA) was used to design and conduct the interviews. Data were obtained on demographics, drug use behaviors and overdose experiences, substance use treatment history, COVID-19 infection and vaccination, HIV and HCV self-reported status and treatment history, and other factors. We also asked about having “chronic conditions” (diabetes, overweight, heart diseases, and lung disease/breathing disease) that may increase the risk of severe COVID-19 disease.

We included a section on attitudes towards COVID-19 vaccination. The COVID-19 attitudes, beliefs, and knowledge scale was constructed from 16 items that were each scored from 1 to 4 (Strongly Agree, Agree, Disagree, and Strongly Disagree), with reverse coding for specific items as shown in Table 1 [19]. Across our sample, composite scale scores ranged from 16 to 64. Lower scores reflected positive attitudes toward vaccination or agreement with evidence-based public health approaches to the COVID-19 pandemic (“pro-vaccine”). Reliability of the scale was more than acceptable (Cronbach’s α = 0·81).

**Table 1:** Anti-Vaccine Attitudes Scale

Item—Positively scored
It is important to get a lot of people vaccinated so that we can go back to normal life Overall, the U.S. government has handled the COVID-19 pandemic well, for its citizens Overall, the Chinese government has handled the COVID-19 pandemic well, for its citizens The government should it easier to get vaccinated by providing easy appointments, transportation, and paid time off People who don't get vaccinated risk getting infected and then infecting others I am worried about the new variants to the COVID-19 virus Getting enough people vaccinated so that mask requirements could be reduced was a major accomplishment for the United States
Item—Reverse Scored (r)
I believe that the dangers of COVID-19 have been greatly exaggerated. (r) I do not like vaccines in general. (r) I do not trust pharmaceutical companies. (r) People like me have been mistreated by medical authorities. (r) Even if I got infected, I do not think I would get seriously ill from COVID-19. (r) No one in my family has or is likely to get seriously ill from COVID-19. (r) The economic impact of the lockdowns in the US has been worse than the impact of COVID-19 disease. (r) Mask mandates have been a violation of personal rights. (r) The vaccines were developed too quickly to know if they are safe. (r)

*Scored from 1 (strongly agree), 2 (agree) 3 (disagree) 4 (strongly disagree) for positively scored items. From 1 (strongly disagree), 2 (disagree) 3 (agree) 4 (strongly disagree) for reverse scored items. Lower scores indicate more positive attitudes, higher scores more negative attitudes toward COVID-19 vaccination

Drug toxicology screenings were conducted using the Premier Biotech 13 panel BioCup. HIV, HCV and SARS-CoV-2 antibody testing was done by BioReference Laboratories, using a 4^th^ generation HIV enzyme linked immunosorbent assay (Siemens; Munich, Germany) and a Geenius assay (Bio Rad; Hercules, California USA) with PCR confirmation for HIV-1, a Siemens chemiluminescence assay (Siemens, Munich, Germany) for HCV antibody, and a Roche Elecsys chemiluminescence assay for SARS-CoV-2 (Roche; Geneva, Switzerland).

### Estimating HIV incidence

All participants were asked about previous HIV testing and their HIV status. Participants who reported that they knew they were HIV seropositive before the start of the pandemic and participants for whom we were not able to collect enrollment serum samples were excluded from the seroconversion analysis. Participants who reported that they believed that they were or might have been HIV seronegative at the start of the pandemic and who tested HIV seropositive at study enrollment were considered as likely seroconverters. Participants who tested HIV seronegative at enrollment were considered to have avoided HIV infection during the pandemic, and their time at risk was the time from the start of the pandemic in March 2020 to the time of their enrollment HIV testing.

### Missing data

There were very few missing data. If data were missing for a particular analysis, subjects with missing data were excluded from that analysis, unless otherwise noted. Many of our respondents had been injecting for decades and had damaged/collapsed veins, which made it difficult for even our very experienced phlebotomists to draw sufficient blood. Thus, we had occasional “quantity not sufficient” for serology results.

### Honoraria

Participants received $30 for completing the baseline interview, $50 for the 6-month follow-up interview and $10 for each person, up to six, that they successfully recruited to the study.

## Results

### Characteristics of the sample

Table 2 presents the summary statistics of the demographics, COVID-19 vaccination, drug use behaviors, and mental well-being of the study sample. Among the 275 eligible PWID participants who completed a baseline questionnaire and specimen testing, the mean age was 49 ± 11 years, ranging from 23 to 70 years. Approximately three-quarters (71%) of the participants were male, 35% were Black, 26% were Hispanic, and 69% had received high school, GED, or higher level of education. A majority of the participants reported that their main source of income in the past six months was government benefits (71%) and 42% reported being unstably housed or homeless in the past six months. Sixty-five percent of the participants reported having experienced food insecurity in the last 6 months.

**Table 2:** Baseline characteristics of Participants by receiving one shot of covid vaccination, *N* = 275, data collected October 5th, 2021 to September 13th, 2022

Variable		Received COVID-19 vaccination		
	Overall, *N* = 275	Not vaccinated, *N* = 53	Vaccinated, *N* = 222	*p*-value
*Demographics*				
*Gender (%)*				0.39
Male	194 (71%)	34 (64%)	160 (72%)	
Female	80 (29%)	19 (36%)	61 (27%)	
Transgender	1 (0%)	0 (0%)	1 (0%)	
Mean age (SD)	49(11)	48(12)	49(10)	0.60
*Race or ethnicity (%)*				0.28
Non-Hispanic White	83 (30%)	13 (25%)	70 (32%)	
Non-Hispanic Black	97 (35%)	22 (42%)	75 (34%)	
Hispanic	71 (26%)	11 (21%)	60 (27%)	
Mixed/Other race	23 (8%)	7 (13%)	16 (7%)	
*Education (Have High school diploma or GED, %)*				0.95
No	84 (31%)	16 (30%)	68 (31%)	
Yes	191 (69%)	37 (70%)	154 (69%)	
*Main source of income in last 6 months (%)*				0.69
Regular employment	24 (9%)	5 (9%)	19 (9%)	
Government benefits	194 (71%)	35 (66%)	159 (72%)	
Irregular employment or friend/relative’s income	31 (11%)	6 (11%)	25 (11%)	
Possibly illegal	26 (9%)	7 (13%)	19 (9%)	
*Housing status in last 6 months (%)*				0.71
Stably housed	101 (37%)	20 (38%)	81 (36%)	
Housed with friends/relatives	58 (21%)	13 (25%)	45 (20%)	
Unstable/homeless	116 (42%)	20 (38%)	96 (43%)	
*Experienced food insecurity in last 6 months (%)*				0.29
No	97 (35%)	22 (42%)	75 (34%)	
Yes	178 (65%)	31 (58%)	147 (66%)	
*Have a smartphone*				0.90
No	43 (16%)	8 (15%)	35 (16%)	
Yes	232 (84%)	45 (85%)	187 (84%)	
*COVID-19 vaccination and comorbidity*				
* Fully vaccinated (%)*				
No	65 (24%)	53 (100%)	12 (5%)	
Yes	210 (76%)	0 (0%)	210 (95%)	
*COVID-19 antibody test*^*1*^* (%)*				< 0.001
Negative	36 (13%)	19 (36%)	17 (8%)	
Positive	238 (87%)	34 (64%)	204 (92%)	
*Mean vaccination attitude score (SD)*	25(6)	31(5)	24(6)	< 0.001
*Number of pre-existing conditions*^*2*^				0.85
None	154 (56%)	28 (53%)	126 (57%)	
1	90 (33%)	19 (36%)	71 (32%)	
2–4	31 (11%)	6 (11%)	25 (11%)	
*Drug use history*				
*Main drug (%)*				0.96
Heroin	222 (81%)	44 (83%)	178 (80%)	
Cocaine	31 (11%)	5 (9%)	26 (12%)	
Other drugs	22 (8%)	4 (8%)	18 (8%)	
*Frequency of use for main drugs in last 6 months (%)*				0.26
Once a week or less	15 (5%)	1 (2%)	14 (6%)	
Several times per week	82 (30%)	13 (25%)	69 (31%)	
Daily or more frequently	177 (65%)	39 (74%)	138 (62%)	
*Receiving methadone maintenance treatment (%)*				0.90
Never	55 (20%)	12 (23%)	43 (19%)	
Previous	72 (26%)	13 (25%)	59 (27%)	
Current	148 (54%)	28 (53%)	120 (54%)	
*Used methadone in last 30 days*				0.08
No	238 (87%)	42 (79%)	196 (88%)	
Yes	37 (13%)	11 (21%)	26 (12%)	
*Mental health*				
*Kessler Psychological distress (%)*				0.70
Moderate/Minor	170 (62%)	34 (64%)	136 (61%)	
Serious	105 (38%)	19 (36%)	86 (39%)	
*Substance use disorder (%)*				0.77
Mild/Moderate	19 (7%)	4 (8%)	15 (7%)	
Severe	256 (93%)	49 (92%)	207 (93%)	

Pearson's Chi-squared test and Fisher's exact test for comparing proportions, Wilcoxon rank-sum test for comparing means. SD: Standard deviation. IQR: Interquartile range

^1^There was one subject whose blood specimen had insufficient quantity due to collapsed vein and no determinate test result of COVID-19 antibody were provided

^2^The chronic conditions surveyed included diabetes, overweight, heart diseases, and lung disease/breathing disease

Many respondents reported substance use problems (65% daily injecting, 93% with severe substance use disorders (SUDs)). About 44% of participants also had one or more chronic condition that would increase the likelihood of developing severe COVID-19.

### Residential geographic distribution of participants

The data were collected at a research site in southern Manhattan. This area, including the Lower East Side and the Village, has been a multi-racial, multi-ethnic area of high drug use since the turn of the twentieth century [20]. The area has excellent public transportation and contains a concentration of services for persons who use drugs (treatment centers, syringe services programs) as well as being a distribution center of illicit drugs. Participants reported residing in 80 of the approximately 140 residential ZIP codes in the city; 36% reported residing in Brooklyn, 24% in Manhattan, 13% in Queens, 10% in the Bronx, 5% in Staten Island, and 12% other or had no regular address.

### COVID-19 vaccination

Among the total sample, 81% (222 of 275) had received at least one dose of the COVID-19 vaccine. Among persons with at least one immunization, 95% had received two immunizations (were “fully vaccinated”). Only 71 (34% of fully vaccinated and 26% of total sample) reported having received a booster vaccination, but our data collection began in Oct. 2021, and booster authorization for the general adult population occurred shortly after that in November 2021 [21, 22]. SARS-CoV-2 antibody testing showed that 92% were positive among those who reported at least one immunization and 64% tested positive among those who had no immunizations, indicating some loss of antibodies among the vaccinated and a substantial number with antibodies from natural infection.

As shown in Table 2, we also examined the differences in our study sample by vaccination status, whether they received at least one COVID-19 versus no immunizations. There was a highly significant difference in the vaccination attitude scores with a higher score indicating vaccination resistance and being unvaccinated (*p* < 0.001). From our ongoing data collection, we suspect that vaccination attitudes are changing as “COVID-19 fatigue” sets in. We do need, however, to collect data over a longer time period to assess changes in vaccination attitudes and the relationship of attitude changes to obtaining vaccine boosters.

### Drug use and injecting risk behaviors

Table 3 presents data on HIV and HCV serostatus, recent (past 30 days) drug use and HIV risk behaviors by HIV serostatus. There were no statistically significant differences between HIV seronegative and HIV seropositive respondents on any of the variables for which meaningful comparisons could be made. Receptive sharing was low regardless of HIV serostatus. Among the HIV seronegative respondents, approximately 9% reported distributive sharing (passing on syringes that they had used) and 4% reported receptive syringe sharing (injecting with a syringe that had been used by someone else). Among those who were HIV positive, 10% reported receptive sharing, 89% reported being on ART, 89% reported seeing their doctor in the last six months, and among those on ART, 79% reported taking 99% or more of their ART medications on schedule during the previous 6 months. Only one HIV seropositive participant reported distributive sharing, and they reported that they were on ART and were 100% adherent in taking their medication.

**Table 3:** HIV infection and transmission risk factors by baseline HIV test result (*N* = 275)

Variable		Baseline HIV test^1^	
	Overall, *N* = 275	Negative, *N* = 256	Positive, *N* = 19
*Self-reported Last HIV test result (%)*			
Negative	257 (93%)	256 (100%)	2 (11%)
Positive	18 (7%)	0 (0%)	17 (89%)
*Seen doctor for HIV [Last 6 Mon] (%)*			
Yes	17 (6%)	0 (0%)	17 (89%)
Not applicable	258 (94%)	256 (100%)	2 (11%)
*Ever received ART for HIV (%)*			
Yes	17 (6%)	0 (0%)	17 (89%)
Not applicable	258 (94%)	256 (100%)	2 (11%)
*Take HIV meds on schedule [Last 6mon, on a percent scale] (%)*			
50	1 (0%)	0 (0%)	1 (5%)
80	1 (0%)	0 (0%)	1 (5%)
99	1 (0%)	0 (0%)	1 (5%)
100	14 (5%)	0 (0%)	14 (74%)
Not applicable	258 (94%)	256 (100%)	2 (11%)
*Primary source of sterile syringes [Last 30d] (%)*			
Syringe Exchange	148 (70%)	137 (70%)	11 (73%)
Pharmacy	30 (14%)	28 (14%)	2 (13%)
Family doctor/hospital	5 (2%)	5 (3%)	0 (0%)
Drug using family/friends/sex partner	18 (9%)	16 (8%)	2 (13%)
Family or friends who do not use drugs	1 (0%)	1 (1%)	0 (0%)
Buying on the streets (including from drug dealers)	4 (2%)	4 (2%)	0 (0%)
Other	5 (2%)	5 (3%)	0 (0%)
*Median number of new needles obtained [Last 30d] (IQR)*	30 (10, 60)	28 (10, 60)	30 (20, 100)
*Number of people receptive sharing [Last 30d] (%)*			
0	264 (96%)	247 (96%)	17 (89%)
1	6 (2%)	6 (2%)	0 (0%)
2	4 (1%)	3 (1%)	1 (5%)
3	1 (0%)	0 (0%)	1 (5%)
*Number of people distributive sharing [Last 30d] (%)*			
0	252 (92%)	234 (91%)	18 (95%)
1	13 (5%)	13 (5%)	0 (0%)
2	6 (2%)	6 (2%)	0 (0%)
5	3 (1%)	3 (1%)	0 (0%)
10	1 (0%)	0 (0%)	1 (5%)

ART: antiretroviral therapy

^1^There were 23 subjects whose blood specimen had insufficient quantity due to collapsed vein and no determinate test results were provided. The table uses their self-reported last HIV test result, including 1 subject reported HIV positive, and 22 reported HIV negative

The primary locations for obtaining syringes were syringe exchange programs (70%), pharmacies (14%) and from friends, family, or sex partners (9%). The median number of syringes obtained in the last 30 days was 30 (interquartile range: 10–60 syringes). There was no difference in primary sources of syringes or number of new syringes obtained in the last 30 days when PWID were stratified by HIV serostatus.

### HIV transmission

As noted in Methods, we first identified participants who were not at risk for incident HIV infection during the pandemic. Nineteen (7%) participants were HIV seropositive at enrollment. Seventeen of these 19 HIV seropositive participants reported that they had known they were HIV seropositive before the start of the pandemic and that they were receiving ART prior to the start of the pandemic. We thus considered these 17 participants to not have been at risk for incident HIV infection during the pandemic.

There were 2 participants who tested HIV seropositive at enrollment who did not report that they were HIV seropositive at the start of the pandemic. Both participants reported that they had been tested for HIV during the pandemic. One reported that they had tested HIV seronegative and the other reported that they did not know their test results. (It is not unusual for PWID to be tested for HIV and then not return for their test results.) We considered these 2 participants to have seroconverted between the start of the pandemic lockdown and assumed that each of them seroconverted after 1 person-year at risk (halfway between the start of the pandemic lockdown and the times of their enrollment). It is possible that the participant who had tested during the pandemic period but did not know their results actually seroconverted prior to the start of the pandemic period. If this is the case, it would reduce our estimated seroconverion rate by half.

There were 234 participants who tested HIV seronegative at study enrollment. They had a total of 469.15 person-years at risk during the pandemic/lockdown period. Our estimated HIV incidence was 2 probable seroconversions in 518.83 person-years at risk for an estimated incidence rate of 0.39/100 person-years, 95% Poisson CI 0.05–1.39/100 person-years.

## Discussion

### COVID-19 vaccination

NYC made a large effort to encourage vaccination in the general population and focused special efforts on persons likely to be at high risk for severe COVID-19 disease. Our findings regarding vaccination should therefore be considered a result of the respondents’ desires to protect their health and the efforts of the NYC government, many community organizations and many individual health care workers promoting vaccination. For example, multiple participants mentioned health outreach workers who provided vaccinations in homeless shelters, and our participants who reported unstable housing/being homeless had a (non-significantly) higher rate of vaccination that participants in more stable housing situations. Our participants who reported food insecurity also had a non-significantly higher vaccination rate than the participants who did not report food insecurity.

Considering the many potential barriers PWID faced in receiving the COVID-19 vaccination—lack of employment, unstable housing, severe SUDs–our overall rates of 81% with at least one immunization, and 74% fully vaccinated are moderately encouraging and suggest that the special efforts made in NYC to reach persons at high risk have been effective.

We are aware of two other studies of COVID-19 vaccination among PWID, one in San Diego County, USA [23], and one conducted at syringe service programs in Australia [24]. In both of these studies approximately half of the participants had been vaccinated, but the many differences in methods, dates of data collection, and settings preclude simple comparisons with the present study.

We did not observe a meaningful difference in COVID-19 vaccination by race/ethnicity among our participants. It still may be useful to compare racial/ethnic differences among our PWID participants and the racial/ethnic differences in at least one COVID-19 immunization for adults in NYC as a whole. In our sample: 86% of Whites, 86% of Hispanics/Latinx, and 72% Blacks received at least one immunization compared to 76% of Whites, 95% of Hispanics/Latinx and 73% of Blacks in the city as a whole [25]. Racial/ethnic disparities in COVID-19 are a critical public health problem in the US; here we would note that the disparities we observed do not appear to be worse than the disparities in NYC as a whole.

The biggest obstacle to achieving higher rates of vaccination would appear to be the anti-vaccination attitudes held by a modest but important proportion of our participants. A separate report incorporating these quantitative results and findings from qualitative interviews about vaccine attitudes is in preparation. 

In addition to the 81% of respondents who had received at least one vaccination, 63% of those who had not been vaccinated had antibodies to SARS-CoV-2, for a total of 92% with at least some immunity. We conducted a literature search for other post-delta, post-omicron studies of COVID-19 antibody among unvaccinated persons in NYC, but were not able to locate any such studies.

There is, however, a clear need for continuing efforts to promote COVID-19 vaccination among PWID. New variants arose during our data collection and additional new variants should be expected in the future. On August 31, 2022, an additional booster was authorized for adults that targeted the more recent Omicron BA.4/BA.5 subvariants [26]. Immunity due to vaccination and natural infection will wane over time, requiring booster immunizations. (Note 8% of our vaccinated respondents had lost antibodies to SARS-CoV-2 by the time of study participation.) Continuing efforts to vaccinate PWID will have to occur within a complicated situation of multiple types of health disparities and an environment with considerable mis- and dis-information about COVID-19 and the vaccines. Our early vaccination results presented here do justify some optimism that efforts to provide vaccination to PWID can be successful.

### HIV risk behavior and transmission

A recently developed agent-based model explored injection risk behavior and HIV incidence among PWID in NYC [27]. The model utilized empirical data from 1009 PWID who participated in our research group’s “Risk Factors” study, a long-running serial cross-sectional study of PWID entering substance use treatment programs from 2012 to 2019 [27]. Estimated HIV incidence among PWID in NYC was very low, < 0.5/100 person-years during this period [28]. The two key variables in the model driving low incidence were: 1) an extremely low percentage of the PWID population (< 1%) who were likely to transmit HIV through injecting, i.e., PWID who were HIV seropositive, not on ART, and were engaged in distributive syringe sharing, and 2) a low percentage (5%) of the PWID population who were likely to become exposed to HIV, i.e., they were HIV seronegative and engaged in receptive syringe sharing with relatively large numbers (> 5) of other PWID. The model outputs were compared to multiple empirical estimates of HIV prevalence among PWID in NYC, including a retrospective cohort study conducted within the Risk Factors study [28]. There was close agreement between the ABM model output and the multiple empirical estimates [27]. In this modeling paper, an increase to an HIV incidence of 1/100 person-years was defined as outbreak of HIV among PWID. With the observed incidence rate in this sample, the probability that the “true” HIV incidence was 1/100 person-years or higher was < 0.01.

A comparison of the pre-COVID-19 Risk Factors study data [27] to the current “during COVID-19” data shows very strong similarities:

1. HIV prevalence: Pre-COVID-19: 7%; During COVID-19: 7%
2. PWID likely to transmit HIV—seropositive, not on ART, distributive sharing: Pre-COVID-19: < 1%; During COVID-19: < 1%
3. PWID like to be exposed to HIV—seronegative, receptive sharing with > 5 others: Pre-COVID-19: 5%; During COVID-19: 0%
4. Estimated HIV incidence—from retrospective cohorts: Pre-COVID-19: 0.37/100 PY [28]; During COVID-19: 0.42/100 PY

We attribute the strong similarities between the pre- and during-COVID-19 data to three primary factors: (1) After initial disruptions, syringe service programs rapidly distributed large numbers of sterile syringes, (2) MOUD and ART programs continued to provide services to large numbers of PWID, and in the case of methadone treatment, making treatment more accessible through home delivery and expansion of take-home doses (3), perhaps most importantly, the continued efforts by PWID to practice safer injection.

### Worst case scenario

Given the travel patterns of our participants, it is not difficult to imagine what might happen if sterile syringes and ART were not readily available and PWID had not developed social norms against syringe sharing. PWID would still be coming to Lower Manhattan (and other drug distribution locations) to obtain and use drugs and would be transmitting HIV among themselves. They would then return to their home boroughs where they would also inject and transmit HIV to more PWID. This would generate a true citywide outbreak of HIV among PWID.

### Generalization

The information on restoration/adaption of services for PWID cited in the introduction referred to services in the city as a whole. The data presented here make a strong case that the restoration/adaptation of services has been followed by levels of injecting risk behavior and HIV transmission very similar to those of the pre-COVID-19 “end of the HIV epidemic among PWID” for this sample. This sample was recruited in Lower Manhattan and whether similar levels of risk behavior and transmission have been achieved throughout the city is an empirical question (although our sample includes PWID from all five boroughs of NYC). If similar levels have not been achieved, it would be critical to determine why not. This would also apply to other cities that had very low risk behavior and incidence prior to COVID-19. As PWID who are unstably housed are at particularly high risk for outbreaks of HIV [29], research might focus on this group to identify post-COVID-19 outbreaks.

Generalization from data collected during a pandemic situation clearly must be done with caution. Large numbers of our participants reported engaging in HIV prevention and care services, 80% in syringe access programs (70% using syringe services and 10% using pharmacy sales) as their main source of sterile syringes, 54% receiving methadone, and over 90% of the HIV seropositive PWID receiving ART. Thus, we believe that our results would be most likely to generalize to other groups of PWID with high rates of prevention and care service utilization, and least likely to generalize to groups of PWID who were not utilizing HIV prevention and care services during the pandemic.

### Limitations

There are several limitations that should be noted. First, while we utilized RDS methods for recruiting subjects, we were interrupted by the Omicron surge and limited by COVID-19 protocols at the research site, so that we considered our sample to be a convenience sample. We did not use RDS weighting because we suspect that the assumptions upon which RDS is based would not apply to social interactions among PWID during the pandemic. Second, public health guidance (stay at home, avoid crowded situations, maintain social distance) probably reduced willingness to participate in the study. Note that there was a general reluctance of people in the city as whole to visit healthcare facilities during the pandemic. Third, there were not enough participants reporting injecting risk behavior or seroconverting for meaningful statistical analysis of these variables.

Any survey that asks sensitive questions about topics such as illicit drug use and HIV risk behavior needs to be concerned about social desirability bias. There is a possibility that HIV risk behavior and HIV seropositive status were underreported due to social desirability bias, and a possibility that social desirability might also lead participants to report that they were HIV seronegative when they were actually seropositive at study entry. However, the low levels of injecting risk behavior and the low rate of estimated HIV incidence are epidemiologically consistent.

## Conclusions

Compared to the general population, PWID typically experience many social, economic and health disadvantages. The stresses of the COVID-19 pandemic and its lockdowns have the potential to increase the multiple problems experienced by PWID including developing severe COVID-19 disease, increased substance use problems, and increased HIV risk behavior leading to HIV transmission. The participants in this study clearly had substance use problems and severe economic difficulties. The responses of the PWID in this study to the COVID-19 pandemic, however, can be characterized as “resilient” and “pro-health.” A large majority were vaccinated, and the group, as a whole, maintained the very low rates of injecting risk behavior and HIV transmission observed in NYC prior to the pandemic.

## Data Availability

The data supporting the findings of this study contains Personal Health Information (PHI) which is protected under the Health Insurance Portability and Accountability Act (HIPPA), such as vaccination status and whether the individual suffers from any of the “underlying conditions” that would be likely to make a COVID-19 infection more severe. Access to the data can be provided through an approved Data Use Agreement between our institution (New York University) and the institution with which the user is affiliated. Persons wanting to access the data should communicate with the NYU IRB (email contact:irbinfo@nyulangone.org) to initiate a Data Use Agreement.

## Abbreviations

ART: Antiretroviral therapy
CI: Confidence interval
GED: General education development
HCV: Hepatitis C
HIV: Human immunodeficiency virus
IQR: Interquartile Range
IRB: Institutional review board
MOUD: Medication for opioid use disorder
NYC: New York City
PWID: Persons who inject drugs
PWUD: Persons who use drugs
PY: Person Years
RDS: Respondent driven sampling
SD: Standard deviation
SSP: Syringe service providers
SUD: Substance use disorder
USA: United States of America
